# Prescreening for osteoporosis with forearm bone densitometry in health examination population

**DOI:** 10.1186/s12891-022-05325-6

**Published:** 2022-04-22

**Authors:** Chun Yue, Na Ding, Lu-Lu Xu, Ya-Qian Fu, Yuan-Wei Guo, Yan-Yi Yang, Xian-Mei Zhao, Zhi-Feng Sheng

**Affiliations:** 1 Health Management Center, The Second Xiangya Hospital of Central South University, Changsha, Hunan China; 2 National Clinical Research Center for Metabolic Diseases, Hunan Provincial Key Laboratory of Metabolic Bone Diseases, Department of Metabolism and Endocrinology, Health Management Center, The Second Xiangya Hospital of Central South University, Changsha, Hunan China

**Keywords:** Bone mineral density, Forearm bone densitometry, Prescreening, Osteoporosis

## Abstract

**Background:**

Early detection and timely prophylaxis can retard the progression of osteoporosis. The purpose of this study was to determine the validity of peripheral Dual Energy X-ray Absorptiometry (DXA) test for osteoporosis screening. We examined peripheral bone mineral density (BMD) using AKDX-09 W-I DXA densitometer. Firstly, we acquired BMD data from manufacturer-supplied density-gradient phantoms and 30 volunteers to investigate its accuracy and precision, then we measured BMD for 150 volunteers using both AKDX (left forearm) and Hologic Discovery Wi (left forearm, left hip and L1 - L4 vertebrae) simultaneously. Correlation relationship of BMD results acquired from two instruments was assessed by simple linear regression analysis, the Receiver Operating Characteristic (ROC) curves and Areas Under the Curves (AUCs) were evaluated for the diagnostic value of left forearm BMD measured by AKDX in detecting osteoporosis.

**Results:**

In vitro precision errors of AKDX BMD were 0.40, 0.20, 0.19%, respectively, on low-, medium-, and high-density phantom; in vivo precision was 1.65%. Positive correlation was observed between BMD measured by AKDX and Hologic at the forearm (*r* = 0.670), L1–L4 (*r* = 0.430, femoral neck (*r* = 0.449), and total hip (*r* = 0.559). With Hologic measured T-score as the gold standard, the sensitivity of AKDX T-score < − 1 for identifying suboptimal bone health was 63.0 and 76.1%, respectively, at the distal one-third radius and at any site, and the specificity was 73.9 and 90.0%, respectively; the AUCs were 0.708 and 0.879. The sensitivity of AKDX T-score ≤ − 2.5 for identifying osteoporosis at the distal one-third radius and at any site was 76.9 and70.4%, respectively, and the specificity was 80.4 and 78.0%, respectively; the AUCs were 0.823 and 0.778.

**Conclusions:**

Peripheral DXA appears to be a reliable tool for prescreening for osteoporosis.

**Supplementary Information:**

The online version contains supplementary material available at 10.1186/s12891-022-05325-6.

## Introduction

Osteoporosis is characterized by low bone mineral density and microarchitectural deterioration of bone tissue and consequent proneness to debilitating fragility fractures. The prevalence of osteoporosis and the incidence of fragility fracture have increased markedly over the last three decades in China [1]. In 2018, China’s first large-scale multicenter epidemiological survey of osteoporosis showed a osteoporosis prevalence of 32.1% in women and 6.0% in men aged > 50 years, which translates into an estimated population of 49.3 million and 10.9 million, respectively [2]. Osteoporotic fracture is a serious disabling condition with high mortality rate and so imposes a heavy burden on the family, society, and medical resources. It is estimated that 5.99 million osteoporotic fractures will occur in 2025 in China, which is a 2.7-fold increase since 2010, and that will cost the country $25.43 billion [3].

Osteoporosis is preventable and treatable, but only a small proportion of high-risk individuals are evaluated and treated. The National Osteoporosis Foundation treatment guidelines and the World Health Organization (WHO) recommend assessment of bone mineral density (BMD) by dual-energy x-ray absorptiometry (DXA) as the gold standard for osteoporosis screening [4–6]. At many centers, hip and spine BMD measured by DXA is used to assess risk of hip fracture and monitor treatment efficac y[7, 8]. But axial DXA examinations is restricted to a few large centers because the equipment is dependent on imports and the large volume requires a dedicated room; moreover, the high testing cost and the long testing time makes axial DXA unsuitable for use during routine physical examination and for screening of large groups. According to the 2013 International Osteoporosis Foundation (IOF) Asia Pacific Audit report, access to DXA is limited in China, with only 0.46 DXA systems available per million citizens [9]. Cheaper and more convenient methods of evaluating BMD are needed, and peripheral DXA devices might be the answer [10].

BMD measurements at the forearm have been validated for osteoporosis diagnosing and follow-up evaluations [11]. The incidence of osteoporotic forearm fractures is increasing,[12, 13] and forearm BMD has been shown to be a better predictor of forearm fracture risk than BMD at other skeletal site s[14, 15] as it best reflects cortical bone damage. Peripheral DXA is portable, inexpensive, time saving, and well suited for osteoporosis and fracture screening in large populations. Prescreening with peripheral DXA may even help with reducing unnecessary axial DXA measurement in individuals with high BMD and low fracture risk.

In this study, we used a locally made peripheral DXA device (AKDX-09 W-I), with a linear double-energy X-ray sector beam scanning mode, low radiation exposure, and short scanning time (no more than 5 s) for measurement of distal forearm BMD. The purpose of the study was to verify the accuracy of the AKDX by comparison with axial DXA (performed with a Hologic DXA system) and to evaluate its effectiveness in osteoporosis screening.

## Methods

### Participants

The study participants were selected from individuals coming to the Health Management Center of Second Xiangya Hospital, Central South University, Hunan province, China, for routine health examination between February and April 2021. Participation in the study was voluntary. The exclusion criteria were 1) under the age of 20, 2) has a history of left forearm, left hip or lumbar fracture, or 3) pregnancy. A total of 150 individuals (38 males and 112 females) met the eligibility criteria. At enrollment, a structured questionnaire was used to collect data on sex, age, age at menopause, medical history, and handedness (ascertained by asking which hand the subject preferred to use for the majority of tasks).

Height and weight were recorded by well-trained examiners. The subject took off his or her shoes, stood on the height and weight meter (SK-X80, Shuangjia, Shenzhen, China) in a single shirt, raised his chest and looked straight ahead. Height is expressed as centimeters (cm) and is accurate to 0.5 cm. Body weight is expressed as kilograms (kg), accurate to 0.1 kg. BMI was calculated as weight (in kilograms) divided by the height (in meters) squared.

All participants signed an informed consent form prior to enrollment. The study protocol was approved by the Ethics Committee of Second Xiangya Hospital.

### Forearm phantom and BMD measurements

The AKDX forearm phantom was manufacturer-supplied and made of 99% high-purity aluminum in the shape of the radius and ulna. The three-gradient phantom represents low, medium, and high densities with actual BMD values of 0.299 g/cm^2^, 0.494 g/cm^2^, and 0.585 g/cm^2^, respectively. To verify in vitro and in vivo precision, the phantom and the left forearms of 30 healthy volunteers (aged 20–77 years) were scanned twice (with repositioning) on the AKDX (AKDX-09WSh; Shenzhen Xray Electric Co.,Ltd., Shenzhen, China) and the Hologic DXA systems. These 30 volunteers were drawn from the selected 150. There was no significant drift or shift in calibration in the 30 subjects during the study period. Subsequently, the 150 study participants were scanned on each of the two machines. Scans were performed and analyzed by the same well-trained operator according to guidelines provided by the manufacturer.

For AKDX BMD, the complete left forearm was scanned. The AKDX region of interest (ROI) is an area corresponding to the Hologic DXA ultradistal ROI but also covers part of the Hologic DXA mid-distal ROI. The AKDX calibration was checked daily on the aluminum forearm phantom before each scanning session. For Hologic DXA, the complete left forearm, L1–L4, femur and total hip were scanned. The left forearm length (distance between the tips of the olecranon and styloid processes) was measured at frist. The forearm DXA scan defines three ROIs: distal one-third radius, mid-distal, and ultradistal. The Hologic calibration was checked daily using the Hologic calibration phantom and the aluminum spine phantom before each scanning session.

### Statistical analysis

Statistical analysis was performed using SPSS 24.0 (IBM Corp., Armonk, NY, USA). Continuous variables were expressed as the means ± standard deviations. The International Society for Clinical Densitometry (ISCD) DXA Machine Cross-Calibration Tool (https://iscd.org/learn/official-positions/adult-positions/) was used to examine the relationship between mean BMD measured on two machines and to determine the percentage coefficient of variation (CV). Pearson correlation coefficients were calculated to quantify the relationship between AKDX and Hologic measured BMD. Linear regression analyses were performed for AKDX and Hologic measured BMD. The ability of the AKDX to detect osteoporosis was assessed by determining the area under the receiver operating characteristic (ROC) curve. For ROC analysis, the reference value for presence of osteoporosis was as per World Health organization (WHO) criteria, i.e., T-score ≤ − 2.5 either at the distal one-third radius of the nondominant forearm, any site of the distal one-third radius of the left forearm, L1–L4 vertebrae, femoral neck, or total hip. *P* < 0.05 was considered statistically significant.

## Results

### Demographic characteristics

A total of 150 participants (38 males, 112 females; mean age, 48.9 ± 15.9 years; age range, 20–84 years) were included in this study. All participants were right-handed. The mean height was 158.8 ± 7.2 cm and the mean weight was 58.5 ± 9.7 kg. Among 150 participants, a total of 77 postmenopausal women and men over the age of 50. their mean height was 157.4 ± 6.8 cm and the mean weight was 62.0 ± 6.9 kg. The mean BMD at different ROIs varied. Table 1 summarizes the characteristics of the study population.

**Table 1:** Basic Characteristics of the Participants(*n* = 150, 38 males and 112 females)

Variables	Mean ± SD (*n* = 150)	Range (*n* = 150)	Mean ± SD (*n* = 77 of 150)	Range (*n* = 77 of 150)
Age	48.9 ± 15.9	20 ~ 84	62.0 ± 6.9	47 ~ 84
Height (cm)	158.8 ± 7.2	145.0 ~ 184.5	157.4 ± 6.8	145.0 ~ 173.5
Weight (kg)	58.5 ± 9.7	43.8 ~ 101.5	58.5 ± 9.2	45.5 ~ 81.9
BMI (kg/cm2)	23.2 ± 3.0	17.7 ~ 30.1	23.6 ± 2.8	18.5 ~ 30.1
AKDX BMD(g/cm2)	0.406 ± 0.047	0.284 ~ 0.507	0.401 ± 0.053	0.284 ~ 0.531
Hologic distal 1/3 of radius BMD(g/cm2)	0.640 ± 0.087	0.422 ~ 0.842	0.618 ± 0.112	0.074 ~ 0.891
Hologic spine L1–L4 BMD(g/cm2)	0.921 ± 0.145	0.560 ~ 1.350	0.858 ± 0.156	0.556 ~ 1.351
Hologic femoral neck BMD(g/cm2)	0.735 ± 0.128	− 3.3 ~ 4.1	0.678 ± 0.107	0.463 ~ 1.053
Hologic total hip BMD(g/cm2)	0.881 ± 0.132	0.56 ~ 1.35	0.834 ± 0.125	0.559 ~ 1.156
AKDX BMD T score	− 1.401 ± 0.672	−3.24 ~ 0.5	− 1.531 ± 0.720	−3.24 ~ 0.02
Hologic distal 1/3 of radius BMD T score	−1.429 ± 1.195	−4.5 ~ 1.6	−1.854 ± 1.347	−4.5 ~ 1.4
Hologic spine L1–L4 BMD T score	−0.584 ± 1.257	− 3.7 ~ 3.2	− 1.138 ± 1.356	− 3.7 ~ 3.2
Hologic femoral neck BMD T score	−0.863 ± 1.183	−3.3 ~ 4.1	−1.420 ± 0.973	−3.3 ~ 2.0
Hologic total hip BMD T score	−0.155 ± 1.084	−2.8 ~ 3.7	−0.579 ± 0.996	−2.8 ~ 1.9

*BMI* body mass index, *SD* standard deviation, *BMD* bone mineral density

### Measurement precision

Table 2 presents the mean BMD value measured by AKDX and Hologic on the density-gradient phantom and on the forearm of healthy volunteers. There were significant differences between BMD values measured by the two devices. On the low-, medium-, and high-density forearm phantom, the AKDX BMD precision errors were 0.4, 0.2, and 0.19%, respectively; the precision error on the 30 volunteers was 1.65%. The precision errors of Hologic DXA on low-, medium-, and high-density gradient phantom were 0.50, 0.85, and 0.82%, respectively; the precision error in the human volunteers was 1.48%.

**Table 2:** Precision of Measurement--AKDX and Hologic (*n* = 30)

		BMD Mean Value±SD	*p*-value	RMS-SD (g/cm^2^)	Precision (CV%)
Low	AKDX	0.300 ± 0.001	< 0.01	0.033	0.40
	Hologic	0.432 ± 0.003		0.002	0.50
Medium	AKDX	0.492 ± 0.002	< 0.01	0.003	0.20
	Hologic	0.671 ± 0.006		0.006	0.85
High	AKDX	0.582 ± 0.002	< 0.01	0.003	0.19
	Hologic	0.886 ± 0.007		0.007	0.82
Healthy volunteers	AKDX	0.402 ± 0.044	< 0.01	0.019	1.65
	Hologic	0.633 ± 0.072		0.009	1.48

Low, Medium, and High densities gradient phantom, *SD* standard deviation, *RMS-SD* root mean square standard deviation, *% CV* percent coefficient of variation

### Linear correlation coefficients

Table 3 shows the fitted regression equations and the estimated conversion coefficient of slope and intercept with standard error. The Pearson r for correlation between BMD measured by AKDX and Hologic varied from 0.430 to 0.777 according to different measure site of Hologic. The strongest correlation was observed between BMD measured by AKDX and ultradistal radius measured by Hologic (*r* = 0.777), The strongest correlation in the diagnostic site was observed between BMD measured by AKDX the distal 1/3 radius BMD measured by Hologic (*r* = 0.670) and the lowest correlation was observed between BMD measured by AKDX and vertebral BMD measured by Hologic(*r* = 0.430). In addition, there was a good correlation between BMD measured by AKDX and total hip BMD measured by Hologic(*r* = 0.559).

**Table 3:** Linear correlation coefficients(r) of the dual-energy X-ray absorptiometry (DXA) measurements with AKDX to Hologic

Region (BMD, g/cm2)	R	Slope	Intercept	S.E.E. (g/cm2)
ultradistal radius	0.777	0.536	0.187	0.031
distal 1/3 of radius	0.670	0.400	0.150	0.041
Spine L1–L4	0.430	0.170	0.250	0.012
Femoral neck	0.449	0.180	0.280	0.011
Total hip	0.559	0.220	0.210	0.011

*R* Correlation coefficient, *S.E.E* Standard errors of the estimate

**Fig. 1:**
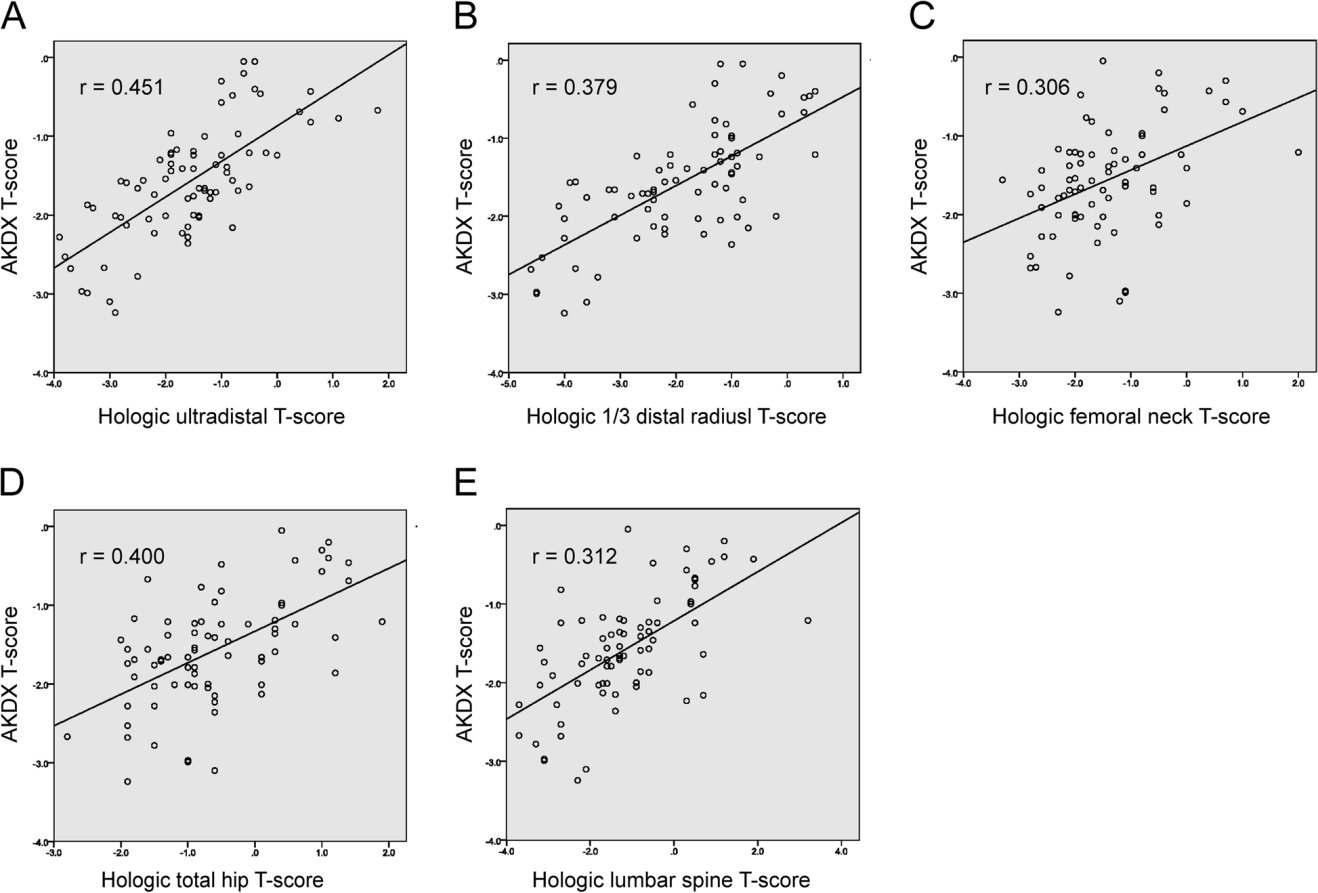
The scatter plots of AKDX T-scores and DXA T-scores

Figure 1 shows the distribution and correlations between AKDX T-scores and Hologic.

DXA T-scores in ultradistal, 1/3 distal radius, total hip, femoral neck, and lumbar spine. AKDX T-scores and Hologic DXA T-scores at all the skeletal sites had significant positive correlation (*r* = 0.306–0.451; *p*<0.001). AKDX T-scores showed the highest correlations with Hologic DXA T-scores in ultradistal among all measured sites.

### ROC analysis

**Fig. 2:**
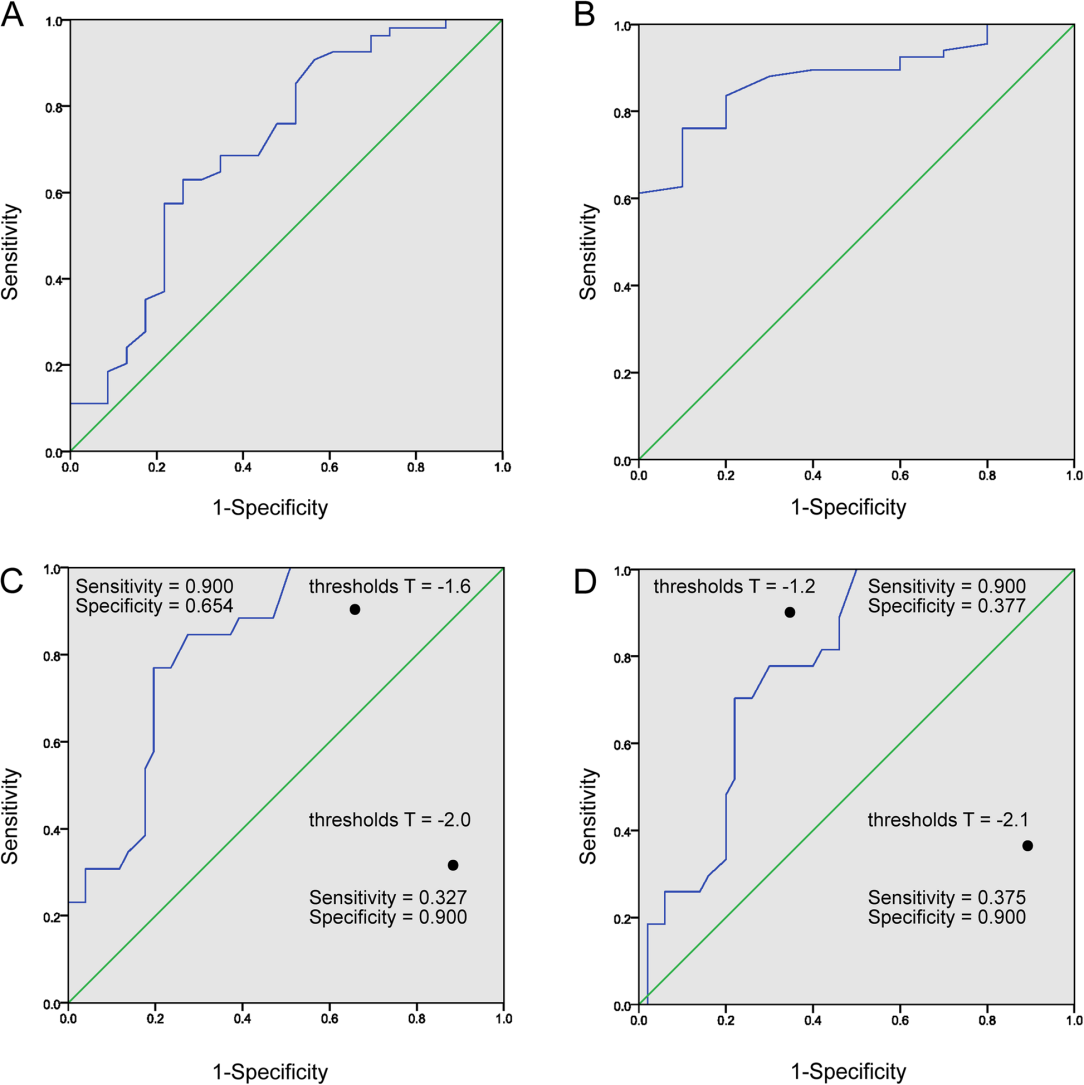
Receiver operating characteristic curves of AKDX in identifying the risk of osteoporosis with reference to Hologic DXA. **A, B** Receiver operator characteristic curves on the performance of AKDX (AKDX T score < − 1) in identifying subjects at risk of suboptimal bone health (Hologic DXA T score < − 1), (**A**) AKDX forearm distal VS Hologic distal 1/3 of radius, (**B**) AKDX forearm distal VS Hologic any site. **C, D** Receiver operator characteristic curves on the performance of AKDX (AKDX T score ≤ − 2.5) in identifying subjects at risk of suboptimal bone health (Hologic DXA T score ≤ − 2.5), (**C**) AKDX forearm distal VS Hologic distal 1/3 of radius, (**D**) AKDX forearm distal VS Hologic any site. Any site: distal one-third radius, L1–L4 vertebrae, femoral neck and total hip

The performance of AKDX was assessed through ROC analysis, with Hologic as the reference (Fig. 2A-D). Among 150 participants, a total of 77 postmenopausal women and men over the age of 50 were recruited. According to WHO diagnostic criteria, a BMD T-score ≤ − 2.5 indicates osteoporosis, while a T-score between − 1 and − 2.5 indicates osteopenia [16]. The sensitivity of AKDX T-score < − 1 for identifying suboptimal bone health (Hologic DXA T-score < − 1) at the distal one-third radius of the non-dominant forearm and at any site (i.e., distal one-third radius, L1–L4 vertebrae, femoral neck, and total hip) was 63.0 and 76.1%, respectively, and the specificity was 73.9 and 90.0%, respectively; the AUCs were 0.708 and 0.879. The number of Hologic DXA T-score < = − 1 at the forearm and at any site were 53 and 66, respectively. The sensitivity and specificity provided for AKDX T-scores of − 1 for identifying suboptimal bone health (Hologic DXA T-score < − 1) at the distal one-third radius were 0.885 and 0.435, and at any site were 0.877 and 0.667(Table 4). The sensitivity of AKDX T-score ≤ − 2.5 for identifying osteoporosis (Hologic DXA T-score ≤ − 2.5) at the distal one-third radius and at any site were 76.9 and 70.4%, respectively, and the specificity was 80.4 and 78.0%, respectively; the AUCs were 0.823 and 0.778. The number of Hologic DXA T-score < = − 2.5 at the forearm and at any site were 23 and 25, respectively, The sensitivity and specificity provided for AKDX T-scores of − 2.5 for identifying suboptimal bone health (Hologic DXA T-score ≤ − 2.5) at the distal one-third radius were 0.348 and 1, and at any site were 0.292 and 0.981. AKDX T-score thresholds correspond to 90% sensitivity at the distal one-third radius was − 1.6, and it was − 1.2 at any site. AKDX T-score thresholds correspond to 90% specificity at the distal one-third radius was − 2.0, and it was − 2.1 at any site (Table 5).

**Table 4:** The performance of AKDX in identifying subjects with suboptimal bone health (Hologic DXA T-score < −1)

	Sen.(%)	Spe.(%)	AUC	95% CI	*p*-value
distal 1/3 of radius (*n* = 53)	0.630	0.739	0.708	0.573 ~ 0.843	0.004
Any site (*n* = 66)	0.761	0.900	0.879	0.792 ~ 0.967	<0.001
AKDX T-scores of −1 (distal 1/3 of radius)	0.885	0.435			
AKDX T-scores of −1 (Any site)	0.877	0.667			

Significant *p-values* are italicized. *AUC* area under curve, *CI* confidence interval, *Sen.* sensitivity, *Spe.* specificity, Any site, any site of the distal 1/3 of radius of left forearm, spine L1 - L4, femoral neck, total hip

**Table 5:** The performance of AKDX in identifying subjects with osteoporosis (Hologic DXA T-score ≤ −2.5)

	Sen.(%)	Spe.(%)	AUC	95% CI	*p*-value
distal 1/3 of radius (*n* = 23)	0.769	0.804	0.823	0.732 ~ 0.915	< 0.001
Any site (*n* = 25)	0.704	0.780	0.778	0.677 ~ 0.880	< 0.001
AKDX T-scores of −2.5 (distal 1/3 of radius)	0.348	1			
AKDX T-scores of −2.5 (Any site)	0.292	0.981			
AKDX T-scores of −1.6 (distal 1/3 of radius)	0.900	0.654			
AKDX T-scores of −1.2 (Any site)	0.900	0.377			
AKDX T-scores of −2.0 (distal 1/3 of radius)	0.327	0.900			
AKDX T-scores of −2.1 (Any site)	0.375	0.900			

Significant *p-values* are italicized. *AUC* area under curve, *CI* confidence interval, *Sen.* sensitivity, *Spe.* specificity. Any site, any site of the distal 1/3 of radius of left forearm, spine L1 - L4, femoral neck, total hip

## Discussion

In this study, the precision of AKDX was verified on the density-gradient phantom provided by the manufacturer and by two repeated measurements of BMD in healthy volunteers. The validity of peripheral AKDX for prescreening for osteoporosis was assessed by comparing with axial Hologic DXA. The locally made peripheral AKDX machine was found to be a feasible and precise modality for osteoporosis prescreening.

This study successfully proved the validity of portable peripheral AKDX DAX densitometer in BMD measurement in adult physical examination population. Pearson correlation analysis demonstrated that AKDX measured forearm BMD was significantly correlated to Hologic measured BMD at distal one-third of radius, total hip, femoral neck, and L1–L4. The strongest correlation was observed between AKDX measured forearm BMD and Hologic measured ultradistal radius BMD. And the strongest correlation in the diagnostic site was observed between BMD measured by AKDX the distal 1/3 radius BMD measured by Hologic. This finding is in conformity with previous studies which were conducted in young children and adults and demonstrated the consistency between peripheral BMD and Hologic measured forearm BMD [17, 18]. Better comparability of forearm densitometry would require, in particular, standardization of the ROIs to be used. It should be noted that the ratio of cortical to trabecular bone mass increases from the proximal to the distal forearm [19], so mismatch of ROIs could result in different rates of loss or gain. Thus, when individuals in clinical practice are being followed up over time, change of device should be avoided.

Assessment of precision errors in BMD is a prerequisite. Without knowing the precision of BMD measurement, it is not possible to accurately assess changes that occur over time. Precision refers to the reproducibility of results when quantitative measurements are repeated [20]. According to the methodology recommended by the International Society for Clinical Densitometry (ISCD) [4], precision is evaluated by repeated measurement on the manufacturer-supplied phantom (in vitro) or on a patient (in vivo), and is expressed as the percentage coefficient of variation. Precision errors of bone densitometry techniques are remarkably low, much lower than that of many other quantitative measurements used in clinical medicine. In our study, the density phantom CVs were all below 1%, the vivo CV were below 2%, referring to the Table 1.The reported CVs of most DXA instruments are about 0.5–1% in vitro and 1–2% in vivo [21]. In previous reports, the CVs were 0.5–3% for central DXA test and was 1% for peripheral DXA test [21–23]. DXA measurements at the radius or ulna showed precision errors in vivo of 0.5–1.9% [24, 25]. Our results showed that the precision of peripheral DXA measurement was similar to that of other clinically accepted densitometry techniques, which was acceptable. The precision of BMD measurement must be maintained at the highest level so that biological changes can be detected as early as possible. Our study indicated that the AKDX instrument had sufficient precision for screening for osteoporosis.

This study evaluated the ability of peripheral AKDX to identify osteoporosis and low bone mass was assessed. In evaluating the peripheral device, we first used the T-score derived from the database supplied by the manufacturer, which is standard practice for health care professionals using these devices. As recommended by the World Health Organization, osteoporosis was defined as a T-score ≤ − 2.5, while a T-score between − 1 and − 2.5 indicates osteopenia. ROC curve is a useful method to evaluate the value of BMD measurement [26]. In the present study, the area under the curve and the sensitivity and specificity of AKDX (AKDX T-score < − 1) for identifying subjects with suboptimal bone health (i.e., Hologic DXA T-score < − 1) were found to be fair. The sensitivity and AUC of AKDX for detecting osteoporosis (Hologic DXA T-score ≤ − 2.5) in forearm were better than for detecting suboptimal bone health.

BMD measured by peripheral bone densitometry has been shown to be a reliable predictor of fracture risk. Studies using various peripheral bone densitometry instruments have consistently shown that low BMD in the forearm, finger, or heel is associated with high probability of fracture in the lumbar spine, hip, forearm, or ribs within 1 year in postmenopausal white women [14]. Other studies have shown that peripheral BMD together with a thorough clinical evaluation can provide a reliable osteoporosis risk profile and can therefore be used for diagnosis of osteoporosis when central DXA is not available [27].

According to our results, BMD measured by AKDX was significantly correlated to Hologic measures BMD. And the AUC of AKDX in identifying subjects with osteoporosis using T-score measured by Hologic at distal 1/3 of radius and at any site as diagnostic criteria were 0.823 and 0.778, respectively. AKDX would perform well in osteoporosis pre-screening. However, since the site where AKDX measures BMD is not a standard osteoporosis diagnostic site, and no standard is made for osteoporosis diagnosing based on BMD measured by AKDX yet. Also, the application of AKDX is not widespread. The use of AKDX should be limited in screening for individuals who need further DAX test at the present stage, and AKDX is not recommended for osteoporosis diagnosing and therapy monitoring.

This study has several limitations. First, all measurements reported in this study were obtained from AKDX-09 W-I and Hologic DXA scanners. Our findings cannot necessarily be extrapolated to scanners from other manufacturers, as the ROIs could be slightly different for those machines. Second, because of the limited number of participants in this study, we did not attempt subgroup analyses based upon patient characteristics such as age and sex. To find the best cutoff value for identifying osteopenia and osteoporosis and to validate effectiveness of peripheral DXA, larger samples would be required. Third, the target population in our research was a general population with a mean age of 48.9 while the majority osteoporosis patients were postmenopausal women and men over 50 years old. So, the results should be generalized to common osteoporosis population, conservatively, and it should be further verified through expanding sample size in the future. Nevertheless, the information from this study can serve as a guide for future studies using AKDX for screening of bone health.

## Conclusions

Peripheral BMD measurement with the AKDX-09 W-I appears to be a feasible and reliable method for prescreening for osteoporosis diagnosis. Because of its portability, low cost, and ease of use, the AKDX is particularly well suited for osteoporosis screening in rural areas or in patients who are home bound. Clinical significance and cost-effectiveness of peripheral DAX screening should be further studied in the future.

## Supplementary Information

**Additional file 1**

## Data Availability

The datasets generated and/or analysed during the current study are not publicly available due to volunteers and patients’ privacy but are available from the corresponding author on reasonable request.

## Abbreviations

DXA: Dual Energy X-ray Absorptiometry()
BMD: Bone mineral density
ROC: Receiver Operating Characteristic
AUCs: Areas Under the Curves
IOF: International Osteoporosis Foundation
ISCD: International Society for Clinical Densitometry
ROI: Region of interest
CV: Coefficient of variation
