# Supplementary material for: Prescreening for osteoporosis with forearm bone densitometry in health examination population
Source: BMC Musculoskelet Disord. 2022 Apr 22;23:377. doi: 10.1186/s12891-022-05325-6 (PMC9027342; doi:10.1186/s12891-022-05325-6)
Supplement: Supplementary file 1 — Additional file 1 [file 12891_2022_5325_MOESM1_ESM.docx]

Supplementary material

**Methods**

the calculation for in vitro and in vivo precision

For the calculation in vivo precision, we recruited 30 right-handed (aged 20-77 years) healthy individuals to measure left forearm BMD on AKDX and Hologic DXA on the same day, and each person was scanned twice (with repositioning). Then we import the bone mineral density results into the ISCD Advanced Precision Calculating Tool to calculate the root mean square standard deviation (RMS-SD) and Coefficient of Variation (precision).

For the calculation in vitro precision, the density gradient phantom provided by the company was used. The AKDX forearm phantom was manufacturer-supplied and made of 99% high-purity aluminum in the shape of the radius and ulna. The three-gradient phantom represents low, medium, and high densities with actual BMD values of 0.299 g/cm2, 0.494,g/cm2, and 0.585 g/cm2, respectively. With the same person, the same time period and the constant indoor temperature, density gradient bulk mode was scanned three times (with repositioning) on the AKDX and hologic DXA for 30 consecutive days. The BMD results were then imported into the ISCD Advanced Precision Calculating Tool to calculate the root mean square standard deviation (RMS-SD) and Coefficient of Variation (precision) for the group.


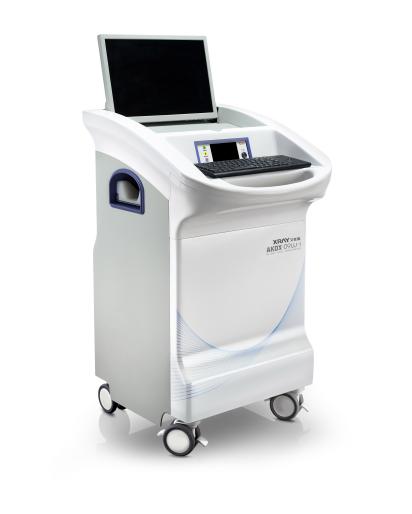


Figure 1 The photo of the portable instrument AKDX


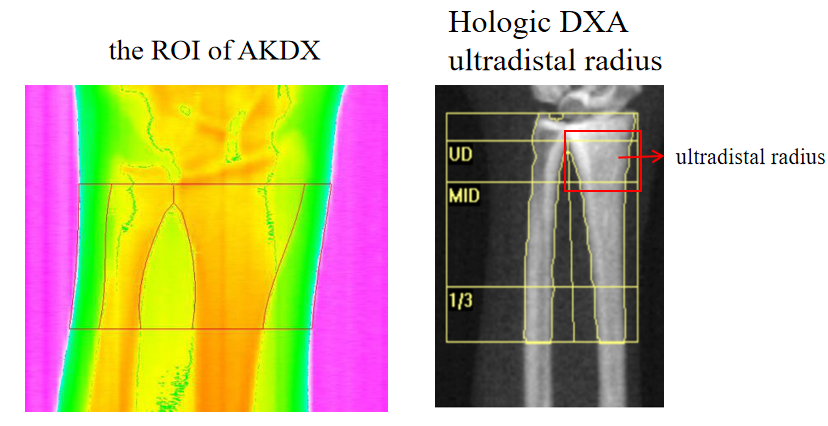


Figure 2 The picture of the ROI of AKDX and Hologic DXA ultradistal radius

Table 1 The agreement between AKDX and DXA

|  | | k | *p-*value |
| --- | --- | --- | --- |
| suboptimal bone health | distal 1/3 of radius | 0.405 | <0.001 |
|  | Any site | 0.448 | *<0.001* |
| osteoporosis | distal 1/3 of radius | 0.428 | *<0.001* |
|  | Any site | 0.428 | *<0.001* |

Significant p-values are italicized; κ, kappa coefficient

Table 1 Kappa statistics revealed that the agreement for osteoporosis risk stratification between AKDX and DXA was moderate
